# Hippocampal harms, protection and recovery following regular cannabis use

**DOI:** 10.1038/tp.2015.201

**Published:** 2016-01-12

**Authors:** M Yücel, V Lorenzetti, C Suo, A Zalesky, A Fornito, M J Takagi, D I Lubman, N Solowij

**Affiliations:** 1 Brain & Mental Health Laboratory, Monash Institute of Cognitive and Clinical Neurosciences, School of Psychological Sciences, Monash University, Clayton, VIC, Australia; 2 Melbourne Neuropsychiatry Centre, Department of Psychiatry, The University of Melbourne and Melbourne Health, Clayton, VIC, Australia; 3 Turning Point, Eastern Health and Eastern Health Clinical School, Monash University, Clayton, VIC, Australia; 4 School of Psychology, University of Wollongong, Wollongong, NSW, Australia; 5 Centre for Health Initiatives, University of Wollongong, Wollongong, NSW, Australia; 6 Illawara Health and Medical Research Institute, University of Wollongong, Wollongong, NSW, Australia

## Abstract

Shifting policies towards legalisation of cannabis for therapeutic and recreational use raise significant ethical issues for health-care providers seeking evidence-based recommendations. We investigated whether heavy cannabis use is associated with persistent harms to the hippocampus, if exposure to cannabidiol offers protection, and whether recovery occurs with abstinence. To do this, we assessed 111 participants: 74 long-term regular cannabis users (with an average of 15.4 years of use) and 37 non-user healthy controls. Cannabis users included subgroups of participants who were either exposed to Δ9-tetrahydrocannabinol (THC) but not to cannabidiol (CBD) or exposed to both, and former users with sustained abstinence. Participants underwent magnetic resonance imaging from which three measures of hippocampal integrity were assessed: (i) volume; (ii) fractional anisotropy; and (iii) *N*-acetylaspartate (NAA). Three curve-fitting models across the entire sample were tested for each measure to examine whether cannabis-related hippocampal harms are persistent, can be minimised (protected) by exposure to CBD or recovered through long-term abstinence. These analyses supported a protection and recovery model for hippocampal volume (*P*=0.003) and NAA (*P*=0.001). Further pairwise analyses showed that cannabis users had smaller hippocampal volumes relative to controls. Users not exposed to CBD had 11% reduced volumes and 15% lower NAA concentrations. Users exposed to CBD and former users did not differ from controls on any measure. Ongoing cannabis use is associated with harms to brain health, underpinned by chronic exposure to THC. However, such harms are minimised by CBD, and can be recovered with extended periods of abstinence.

## Introduction

Despite the promise of cannabis as a therapeutic agent for a number of conditions, long-term, regular cannabis use has been associated with substantial cognitive, mental health and neurobiological harms,^1^ particularly to the hippocampal region.^2, 3^ Yet, there is very little data on whether these brain-related harms are permanent, can recover with abstinence or are influenced by the proportion of Δ9-tetrahydrocannabinol (THC) and cannabidiol (CBD) in the cannabis consumed. Most of the negative effects (for example, impaired cognition, anxiety and psychotic-like experiences)^4, 5^ are attributed to THC, while CBD has ameliorating, antipsychotic/anxiolytic^6, 7^ and potentially neuroprotective properties.^8, 9^

Given the recent legalisation of cannabis in many countries, and ongoing debate regarding its potential impact on public health,^1^ it is critical that we understand whether prolonged exposure to THC is associated with persistent hippocampal harms (or recovers following abstinence) and whether CBD offers some protection.

We sought to address these issues using magnetic resonance imaging (MRI) to compare the hippocampal integrity of long-term, regular cannabis users who were either exposed to THC but not to CBD (CBD group) or exposed to both THC and CBD (CBD+ group), with matched non-using healthy controls. We conducted similar comparisons for regular cannabis users with unknown exposure to CBD (CBDx group), as well as former users who were abstinent for an extended period (Former Users group).

We predicted the following: (i) there would be neurobiological harms associated with long-term and heavy cannabis exposure; (ii) the harms would not be present in individuals who use cannabis containing CBD; and (iii) the harms would recover with abstinence.

## Materials and Methods

### Participants

It is notoriously difficult to recruit samples of long-term and heavy cannabis users without a range of potential confounds (such as psychiatric comorbidity and other drug use) into studies of this type, particularly where extensive MRI scans and other lengthy assessments are required. This strategy often results in smaller sample sizes than may be desired. We have nevertheless recruited to this study 74 well-characterised, extensively screened, psychiatrically healthy individuals with chronic exposure to cannabis (40 males and 34 females) and minimal exposure to other illicit drugs (<50 lifetime occasions; median values for lifetime occasions of use: *n*=0 for benzodiazepines and sedatives, cocaine, inhalants, opiates and other drugs; *n*=5 for amphetamines and ecstasy; and *n*=2 for hallucinogens), from the general community.

Inclusion criteria for all participants were as follows: 19 to 55 years of age; right handedness; English as a first language; normal or corrected-to-normal visual acuity; and ability to travel to the assessment sites. Cannabis users were included if they used cannabis regularly for a minimum of 2 years, with ‘regular use' being defined as at least twice a month; were willing to abstain from cannabis for at least 12 h before attending either assessment session to ensure that examination would occur in a non-intoxicated, non-abstinent state; and were willing to refrain from using substances other than cannabis in the month before assessment. Exclusion criteria for all participants were as follows: presence of neurological disorders or serious head injury; personal psychiatric histories requiring treatment; prolonged use of psychotropic medications; contraindications for MRI (for example, metal implants and claustrophobia); and significant regular use of substances other than cannabis.

Cannabis users were composed of four subgroups (Table 1): (i) current users testing positive for urinary cannabinoid metabolites, but unknown proportional exposure to CBD due to unavailability of hair samples for analysis (CBDx, *n*=19); (ii) current users exposed to THC but not to CBD (from analysis of hair samples reflecting past 3-month exposure; CBD−, *n*=30); (iii) current users exposed to both THC and CBD, ascertained via hair analysis (CBD+, *n*=12); and (iv) former regular cannabis users abstinent by self-report for a mean 29 (s.d.=64) months, with no cannabinoid metabolites detected in hair (*n*=9) or urine (Former Users; *n*=13). (Hair samples were available for eleven former users; nine had no cannabinoid metabolites detected. Two former users had cannabinoid metabolites detected in hair but they had ceased using within the past month and had no metabolites detected in urine. Hair samples were not available for two former users.) Current cannabis use was also confirmed by urinalysis in the users of groups (ii) and (iii). Of note, in current users past-year dosage, measured in cumulative number of cones (that is, 1 joint=3 cones), was significantly and positively correlated with THC levels in hair (*R*=0.35, *P*=0.040). We also recruited 37 non-cannabis using controls (18 males and 19 females), who did not test positive for urinary cannabinoid metabolites. The research protocol was approved by the relevant institutional review boards or ethics committees, and all participants gave written informed consent.

### Substance use measures

We obtained data on past-month substance use via the Timeline Follow-back Procedure,^10^ and comprehensively examined lifetime use of any psychoactive substance other than cannabis using the Substance Use History tool (Orygen Youth Health Research Centre, Melbourne, VIC, Australia.^11, 12, 13, 14^ Alcohol or cannabis use were measured separately via the Alcohol Use Disorder Identification Test^15^ and Cannabis Use Interview, respectively. Results from urine toxicology tests corroborated self-reported substance use.

### MRI protocol and procedures

**Figure 1:**
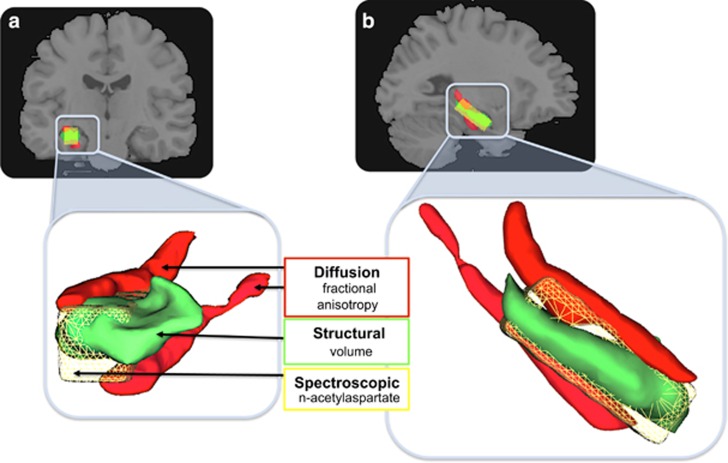
Multimodal assay of the medial temporal grey matter and white matter, and biochemistry. (**a**) Coronal view; (**b**) sagittal view. Concurrent (within subjects) assay of fractional anisotropy from the fimbria and cingulum–hippocampus white-matter fibres (red); anatomical grey-matter volume of the hippocampus proper (green); and *N*-acetylaspartate from a voxel positioned over the hippocampus (yellow). Lower panels show a close-up rendered image to illustrate the relative positions and associations of the medial temporal regions assayed.

Each participant underwent a comprehensive structured interview and psychiatric assessment,^16^ and a series of high-field (3T Siemens TIM Trio, Siemens, Munich, Germany, 32 channel head coil) MRI scans involving structural, diffusion and spectroscopic sequences. From these measures, we derived three well-validated indices of hippocampal integrity: (1) volume; (2) fractional anisotropy; and (3) *N*-acetylaspartate (NAA). These measures provide a detailed assay of hippocampal integrity at the level of macrostructure, microstructure and neurochemistry (Figure 1).

### Structural imaging: volume

Hippocampal volume was measured by manual tracing on T1-weighted structural magnetic resonance images acquired in the sagittal plane with a high-resolution three-dimensional Magnetization Prepared Rapid Acquisition Gradient Echo (MP-RAGE) imaging sequence (Time Repetition=1900 ms, Time Echo=2.15 ms, Field of view=256 mm). Each participant's raw image was bias corrected, skull stripped, rigid-body co-registered to the standard Montréal Neurological Institute template and re-sliced into 1 × 1 × 1 mm^3^ spatial resolution using tri-liner interpolation in SPM8 (Wellcome Trust Centre for Neuroimaging, London, UK). Bilateral hippocampi were traced by an expert (VL), blind to group status, from coronally displayed MRI images using ANALYZE (version 11, Mayo Clinic, Rochester, MN, USA) according to previously validated protocols.^2^ Hippocampal volume was defined as the mean of both left and right hippocampi. Inter-rater and intra-rater reliabilities (VL and SW) were assessed using the intraclass correlation coefficient (absolute agreement) via highly reliable and valid protocols.^2^ In all volumetric measures of the hippocampus, inter-rater and intra-rater reliabilities were consistently intraclass correlation coefficient >0.85. Intracranial volume was calculated by summing the volumes of grey matter, white matter and cerebrospinal fluid, after segmentation in raw images using SPM8, and was then used to adjust hippocampal volumes by the same method described previously by Erickson *et al.*^17^

### Diffusion imaging: fractional anisotropy

Fractional anisotropy was measured in two regions of interest, bilaterally—the fimbria and the hippocampal portion of the cingulum bundle—defined according to the JHU-ICBM white-matter atlas.^18^ The white-matter regions of interest we have considered are based on our previous whole-brain study of axonal disruptions in a large sample of cannabis users.^3^ In this previous study, the fimbria and hippocampal portion of the cingulum bundle were identified as comprising significantly fewer streamlines in the group of cannabis users. As such, these white-matter structures were selected as regions of interest for the present study. Considering additional regions without any *a priori* hypothesis was avoided to reduce the scale of the multiple comparisons problem. Given the sample size, we opted to minimise the number of multiple comparisons by performing inference only on regions for which we had prior evidence for an effect.

Forty-two diffusion-weighted volumes were acquired using a spin-echo echo-planar imaging sequence with the following parameters: *b*-value, 2000 s mm^−1^; 54 consecutive axial slices of 2.3-mm thickness; 104 × 104 image matrix with an in-plane voxel resolution of 2.3 × 2.3 mm; field of view, 24 × 24 cm; repetition time, 7000 ms; echo time, 96 ms; and flip angle, 90°. The diffusion-imaging data were foremost corrected for eddy current distortions by linearly registering each diffusion-weighted volume to the first non-diffusion-weighted volume acquired. Fractional anisotropy images were computed with a least squares fit of the diffusion tensor, carefully inspected for artefacts, warped to Montreal Neurological Imaging standard space and resampled to a 1 × 1 × 1 mm^3^ spatial resolution. Fractional anisotropy values were averaged over all voxels encapsulated by each region of interest to yield a tract-specific measure of white-matter integrity. These steps were implemented using tools in the FMRIB Software Library (www.fmrib.ox.ac.uk/fsl/) with standard options.

### Spectroscopic imaging: *N*-acetylaspartate

**Figure 2:**
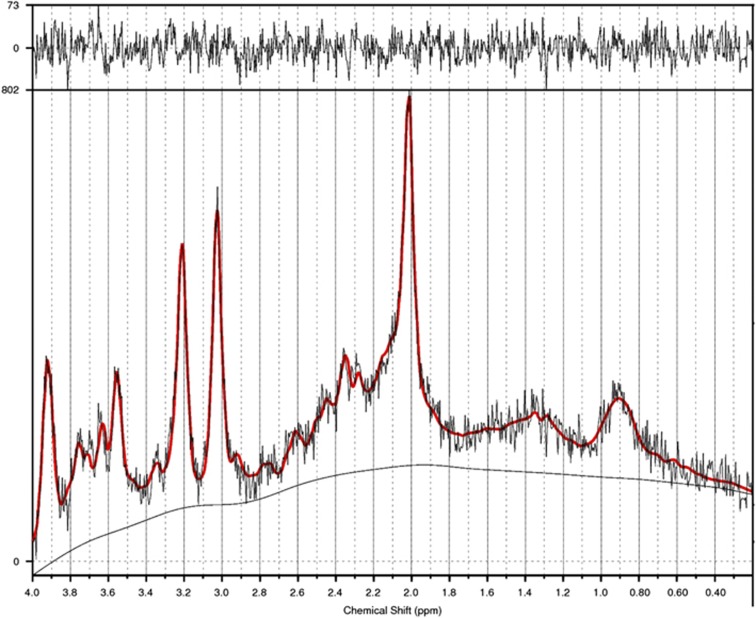
An example of LCModel output for magnetic resonance spectroscopy spectrum from the hippocampus. The red solid line indicates the summary of all fitted metabolites' peaks. For *N*-acetylaspartate (NAA), the major peak is located at 2.02 p.p.m. Mean NAA across all groups (*N*=82) was 6.35 (s.d.=0.97). Separately for each group, mean NAA values were as follows: healthy control, 6.40 (s.d.=0.89); CBDx, 6.28 (s.d.=0.90); CBD−, 5.82 (s.d.=1.11); CBD+, 6.54 (s.d.=0.76); and Former Users, 6.94 (s.d.=0.86).

NAA, a marker of neuronal viability, was acquired from the left hippocampus using a standard short-echo point-resolved magnetic resonance spectroscopy sequence (repetition time 3000 ms, echo time 30 ms, averages 128; with a nominal voxel size ~3 cm^3^ placed to encompass the entire hippocampus). The boundaries of the voxel were, posteriorly, placed ~5 mm anterior to the hippocampus tail; inferiorly, located ~1 mm above the subiculum; and medially, located ~3 slices lateral to the fimbria. We excluded low-quality spectral data (29 out of 111 images) by (i) discarding images with signal-to-noise ratios <6 or broader linewidth, full width at half maximum>0.1 Hz; and (ii) using the commonly accepted Cramer–Rao lower bound criterion of 15% for NAA total to further reject low-quality spectra.^19^ The quantity of excluded images (26%) is in line with expectations in this notoriously difficult brain region to assay.^20^ A sample magnetic resonance spectroscopy spectrum of the hippocampus is displayed in Figure 2.

Of the remaining spectra, the parameters used for this study provided robust signals both for all five groups, and no significant group differences for any measure, with an average signal-to-noise ratio of 9.29 (s.d.=1.46), full width at half maximum of 0.06 p.p.m. (s.d.=0.01) and Cramer–Rao lower bounds of 5.5 (s.d.=1.38). The volume fractions of different tissue types were calculated by initially segmenting T1 images in the raw space using SPM8. Then, a mask (NIfTI format) of the spectroscopic voxel was reconstructed using the dimension, placement and angulation information from magnetic resonance spectroscopy header information. As both images are in the scanner co-ordinates, we co-registered the spectroscopic voxel image to the T1 image and extracted the volume of grey matter, white matter and cerebrospinal fluid within the voxel. The fraction of cerebrospinal fluid was used to correct for the partial volume effect to obtain absolute metabolite tissue concentration in millimole per litre (mM l^−1^). Spectroscopic data were quantified using LCModel (version 6.3, LCMODEL, Oakville, ON, Canada), via a combination of water-suppressed and unsuppressed spectra, to compute the absolute quantification of each metabolite. This was achieved by fitting the experimental spectrum with a group of basis sets, each of which is the spectrum of a specific metabolite or macromolecule.^19^

### Statistical analyses

All analyses were performed using SPSS (version 22.0, IBM, Armonk, NY, USA). All variables met the assumptions of normal distribution and homogeneity by Shapiro–Wilk's and Levene's tests, respectively, enabling the use of parametric tests.

Group comparisons for demographic (age and gender), substance use (alcohol and tobacco), psychopathological symptoms (anxiety and depressive), general functioning, intelligence quotient and gross brain measures (intracranial volume and whole-brain volume) were conducted by a series of *t*-tests (and a *χ*^2^-test for gender). Variables that differed between groups (level of alcohol and tobacco use, anxiety and depressive symptoms, general functioning and intelligence; Table 1) were included, together with age and intracranial volume, as covariates.

**Figure 3:**
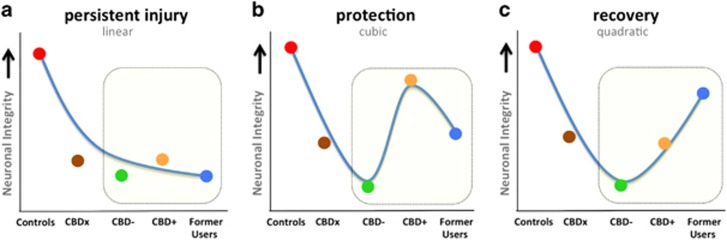
Theoretical models of cannabis-related neurobiological harms. Linear, cubic and quadratic models were tested for each of the three measures of hippocampal integrity. The illustrations are theoretical models of data points that would support each hypothesis of persistence (**a**), protection (**b**) and recovery (**c**). The highlighted window shows the portion of each model to which data from this study contributes new knowledge.

Curve-fitting and polynomial contrasts were applied to the data across the five groups to test the hypotheses that hippocampal harms in regular cannabis users (1) are persistent (linear curve), (2) can be minimised by exposure to CBD but not recovered by abstinence (cubic curve) and (3) can be both minimised by CBD and recovered through long-term abstinence (quadratic curve; Figure 3). Importantly, we tested these models for each of the three measures of hippocampal integrity (volume, fractional anisotropy (FA) and NAA). We hypothesised that in the cannabis group with unknown proportional exposure to THC versus CBD (CBDx) we would replicate our^2^ and others'^21, 22^ previous findings of reduced hippocampal volume in cannabis users and that this group would have reduced neuronal integrity reflected by NAA and FA relative to controls. As such, the CBDx group (presumably reflecting a mixed THC and CBD exposed participants) was placed first in the model after controls. On the basis of previous literature, we predicted the poorest outcomes for the CBD− group, whereas outcomes for CBD+ and Former Users were less predictable. The order of their placement enabled testing of, and distinguishing between, the three modelling functions of persistence, protection and recovery. To correct for multiple comparisons and type I error we used an adjusted critical significance threshold of *P*<0.017 (0.05/3).

To better understand the nature of these findings, we conducted separate analyses of covariance with group as the between-group factor for each hippocampal feature (volume, fractional anisotropy or NAA, respectively), which was used as dependent variable. Significant effects were further investigated using planned pairwise group comparisons, and calculated effect size (ES) (Cohen's *d*) for all between-group analyses.

### *Post hoc* power analysis

G*Power (University of Dusseldorf, Duesseldorf, Germany) was used to conduct a sensitivity analysis for the analysis of covariance analysis. Results showed that a total sample size of 111 enabled power of 0.80 to detect small–medium ES of at least 0.38.

## Results

**Figure 4:**
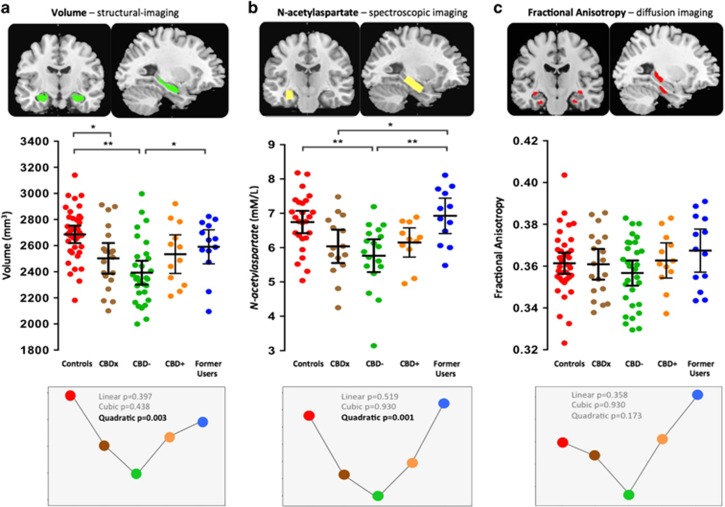
Scatter plots illustrate hippocampal: (**a**) volume; (**b**) *N*-acetylaspartate; and (**c**) fractional anisotropy levels in long-term cannabis users and non-using controls. Controls (*n*=37) are shown in red. Long-term cannabis users (*n*=74) comprise three groups: CBDx, those with unknown exposure to CBD (*n*=19, in brown); CBD−, those known to be exposed to THC but not to CBD (*n*=30, in green); and CBD+, those exposed to both THC and CBD (*n*=12, in orange). Former Users, former long-term cannabis users, abstinent for a mean 29 months (*n*=13, in blue). Horizontal lines represent group means and horizontal bars are the 95% confidence interval. *P<0.05; **P<0.005. Lower panels illustrate the curve-fitting and polynomial contrasts that were applied to the data across the five groups to model our investigational aims.

The results revealed a significant quadratic pattern for both hippocampal volume (*P*=0.003) and NAA (*P*=0.001; Figure 4a and b). Group-wise analyses of covariance for hippocampal volume (a) revealed a main effect of group, F_4, 99_=4.60, *P*=0.002, ES=0.88. *Post hoc* pairwise comparisons showed that relative to controls, the CBDx group had significantly smaller hippocampi (7% reduction, *P*=0.029, ES=0.68) while the CBD− group showed the largest relative reduction in volume (11% smaller, *P*<0.0001, ES>1.0). Former Users did not differ from controls (3% smaller volume in Former Users, *P*=0.26, ES=0.35) and had significantly larger volumes than the CBD− group (8% increase, *P*=0.023, ES=0.79). For hippocampal NAA concentrations (b), there was a main effect of group, F_4, 70_=3.41, *P*=0.013, ES=0.88. *Post hoc* tests showed that NAA levels were significantly reduced in the CBD− group relative to controls (14% smaller, *P*=0.003, ES=0.96) and relative to Former Users (16% smaller, *P*=0.003, ES>1.0). Former Users also had higher NAA levels than the CBDx group (*P*=0.028, ES=0.88) and did not differ from controls (*P*=0.35, ES=0.18). There was no overall significant group difference for hippocampal FA values (main effect F_4, 99_=0.99, *P*=0.42, ES=0.40; c). The ES of all three analyses of covariance (that is, Figure 4a, volume; b, NAA; and c, FA) ranged from 0.40 to 0.88.

Overall, the results support our prediction of reductions in hippocampal integrity in regular cannabis users (Figure 4). The results are also consistent with a protection and recovery model, indicating that cannabis-related hippocampal harms can be minimised by exposure to CBD and recover through abstinence.

## Discussion

We believe this is the first multimodal MRI investigation of prolonged THC and CBD exposure or abstinence on hippocampal integrity in current and former cannabis users, respectively. We confirmed that hippocampal volume is reduced in long-term cannabis users, and found that this atrophy can be restored following prolonged abstinence. Moreover, we show for the first time that both hippocampal volume and neurochemistry are reduced to the greatest extent in users exposed to THC without CBD. In contrast, current users of cannabis containing CBD, as well as former users, show no structural or neurochemical hippocampal differences compared with controls. These findings are consistent with suggestions that CBD may be neuroprotective, perhaps through its role in synaptic plasticity and/or neurogenesis.^23^

The findings have implications for how we conceptualise the long-term effects of cannabis use on the human brain. Our findings suggest that not all cannabis users experience adverse brain and behavioural outcomes^24, 25^ as cannabinoid compounds such as CBD may have a role in minimising harm. Indeed, previous reports have suggested that CBD may ameliorate psychotic and adverse cognitive effects of THC.^6, 21, 26, 27, 28^ For instance, participants pretreated with CBD experience significantly reduced psychotogenic and anxiogenic effects of THC.^26, 27, 28, 29, 30, 31^ At a neural level, CBD exerts opposing effects to THC on brain function and connectivity in regions that are high in cannabinoid receptors including the hippocampus.^27, 29, 30, 31, 32, 33^ Although the relative contribution of THC and CBD to overall brain harm remains unclear, we provide preliminary evidence that these major cannabinoid compounds have a distinct role in hippocampal structural and neurochemical integrity following chronic exposure to cannabis.

The role of CBD in reducing brain and behavioural harms of cannabis warrants consideration when discussing legalisation related to the potency and composition of commercial cannabis. Whether its mandatory inclusion as a public health directive would protect against poor functional outcomes, such as severe mental health problems or cognitive decline among regular cannabis users, remains to be substantiated.

In former users, hippocampal integrity was comparable to controls. This contrasts results from other studies of abstinent former users that have found persistent effects of heavy use on brain function and cognition.^34, 35, 36, 37, 38, 39, 40^ In a prospective study of 1037 people from birth to age 38,^41^ persistent cannabis exposure (assessed at ages 18, 21, 26, 32 and 38 years) was associated with significant decline in neuropsychological performance. Of note, these cognitive impairments did not show significant improvement following reduction of use or complete abstinence (>1 year cessation in some cases). In the context of our data, these findings suggest that functional deficits may persist in abstinent former users, despite apparent recovery of hippocampal integrity.

### Strengths and limitations

The use of hair sample analysis to define groups exposed or non-exposed to CBD is limited in that it only provides information on exposure over the prior 3 months (when 3 cm of hair is analysed). Variable exposure over the lifetime cannot be estimated although it is likely that CBD levels in cannabis were higher in previous decades.^42^ Unfortunately, hair sample analysis was not available for former users, however there was no particular reason for them to overstate their durations of abstinence and hence their self-reports are assumed to be reliable. We also acknowledge the limitations of a cross-sectional design and the modest sample size when examining subgroup differences. It has to be recognised that this is one of the largest samples to date in the international literature and that there are immense difficulties in recruiting abstinent, former users with a long history of regular cannabis use (that is, ~15 years), and without any comorbid substance use and/or mental health problems to complex and demanding studies of this type. At the same time, strict exclusion criteria were also the strength of our study; recruitment of 74 ‘clean' cannabis users enabled our modelling approach to address the gaps in the human literature regarding the neuroprotective and therapeutic potentials of CBD and possible recovery with abstinence.

In conclusion, it seems that CBD and extended abstinence from cannabis may, respectively, protect or restore hippocampal integrity. With ~200 million users worldwide, these findings inform the current debate regarding the legalisation, commercialisation and therapeutic application of cannabis.

## Figures and Tables

**Table 1:** Demographic, substance use, clinical, neuropsychological and brain characteristics

*Group (*n=*111)*	*Controls (*n=*37)*	*Cannabis users (*n=*74)*				*Four-group comparisons*^a^		*Five-group comparisons*^b^	
		*CBDx (*n=*19)*	*CBD− (*n=*30)*	*CBD+ (*n=*12)*	*Former users (*n=*13)*	*F_df_*	P	*F_df_*	P
*Sample characteristics*									
Male/female, %	49/51	47/53	43/57	58/42	85/15	6.71_3, 74_	0.082^c^	6.97_4, 111_	0.138^c^
Age, years	29.95 (11.29)	34.26 (10.96)	32.10 (11.15)	31.67 (10.34)	38.69 (9.80)	1.31_3, 70_	0.276	1.70_4, 106_	0.156^d^
Alcohol, standard drinks per month	19.9 (26.72)	25.96 (31.00)	26.41 (31.03)	8.69 (10.77)	41.62 (28.66)	2.84_3, 70_	0.044	2.53_4, 106_	0.044^d^
Tobacco, cigarettes per week	5.18 (17.95)	48.18 (46.44)	66.97 (54.24)	63.42 (56.79)	24.65 (35.38)	2.40_3, 70_	0.075	10.96_4, 106_	<0.005^d^
Anxiety symptoms	33.73 (7.58)	42.21 (11.71)	38.29 (11.40)	47.33 (12.42)	34.23 (12.49)	2.99_3, 70_	0.037	5.01_4, 106_	0.001^d^
Depressive symptoms	12.17 (2.80)	15.53 (3.45)	13.80 (2.99)	15.50 (3.92)	12.92 (3.09)	2.39_3, 70_	0.076	5.01_4, 106_	0.001^d^
Global functioning	86.76 (4.15)	69.58 (9.01)	76.93 (9.31)	70.67 (10.70)	78.15 (9.04)	3.68_3, 70_	0.016	18.73_4, 106_	<0.005^d^
Intelligence quotient	112.32 (12.72)	103.68 (11.45)	103.17 (10.38)	101.26 (14.78)	109.93 (15.18)	1.24_3, 70_	0.300	3.54_4, 106_	0.009^d^
Intracranial volume, mm^3^	1468.07 (141.02)	1459.49 (124.92)	1401.27 (153.69)	1447.79 (82.74)	1480.47 (121.31)	1.41_3, 70_	0.246	1.32_4, 106_	0.266
Whole-brain volume, mm^3^	1251.02 (120.37)	1245.24 (107.23)	1196.91 (131.85)	1239.19 (69.22)	1257.65 (103.42)	1.24_3, 70_	0.301	1.16_4, 106_	0.333
									
*Cannabis use measures*									
Frequency, days per month									
Lifetime	—	23.1 (7.11)	23.51 (6.78)	26.14 (4.68)	26.77 (8.39)	1.16_3, 70_	0.333	—	
Past 12 months	—	24.31 (9.13)	25.24 (8.8)	24.31 (8.80)	—	0.05_2, 58_	0.954	—	
Dosage, joint cumulative									
Lifetime	—	29,716 (3331)	20,739 (17,907)	27,385 (20,337)	13,151 (12,251)	8.25_3, 70_	0.241	—	
Past 12 months	—	1728 (1401)	1617 (1325)	1623 (917)	—	0.08_2, 58_	0.920		
Duration, years	—	16.95 (9.24)	14.13 (9.53)	15.92 (10.37)	15.69 (9.23)	0.36_3, 70_	0.783		
Onset age, years	—	17.08 (4.36)	16.97 (3.04)	15.58 (2.61)	18.31 (4.03)	1.23_3, 70_	0.305		
THC levels in hair, ng mg^−1^^e^	—	—	0.18 (0.31)	0.27 (0.23)	—	−0.86_33_	0.394		
CBD levels in hair, ng mg^−1^^e^	—	—	—	0.02 (0.01)	—				

Abbreviations: CBD, cannabidiol; THC, Δ9-tetrahydrocannabinol.

All values presented in mean (s.d.).

a Analyses (two tailed) comparing CBDx (those testing positive for urinary cannabinoid metabolites), CBD− (those exposed to THC but not to CBD), CBD+ (those exposed to both THC and CBD) and Former User (former regular users who were abstinent for a prolonged period) groups.

b Analyses (two tailed) comparing controls, CBDx, CBD−, CBD+ and Former User groups.

c *χ*^2^-test.

d Variables used as covariates in all statistical analyses of variance.

e Hair sample analysis (*t*-test) was available only for CBD−, CBD+ and Former User groups. Anxiety (trait) assessed by the State and Trait Anxiety Inventory; depressive symptoms assessed by the Community Assessment of Psychic Experiences; global functioning assessed by the Social and Occupational Functioning Assessment Scale; intelligence assessed using the Wechsler Abbreviated Scale of Intelligence (WASI); and cannabis use patterns assessed by structured interview and Timeline Follow-back Procedures to estimate average lifetime frequency of use (since age of regular use initiation) and cumulative dosage (since first initiation of cannabis), duration of regular use and age of regular cannabis use initiation.
